# Supplementary material for: Frameworks for mitigating the risk of waterborne diarrheal diseases: A scoping review
Source: PLoS One. 2022 Dec 9;17(12):e0278184. doi: 10.1371/journal.pone.0278184 (PMC9733858; doi:10.1371/journal.pone.0278184)
Supplement: S2 Table — (DOCX) [file pone.0278184.s002.docx]

**Table SI3: Complete search strategy PubMed, Scopus and Web of Science**

| **DATA BASE/ SEARCH DATE** | **SEARCH TERMS** | **SEARCH LIMIT** | **RESULTS** |
| --- | --- | --- | --- |
| **PubMed** |  |  |  |
| 13^th^ April 2021 | Waterborne framework | Free search | 147 |
| 13^th^ April 2021 | Waterborne Model | Free search | 1319 |
| 31^st^ August 2021  1 | "diarrh*"[Title/Abstract] | Title/abstract | 116544 |
| 2 | (prevent*[Title/Abstract]) OR (Control*[Title/Abstract]) | Title/abstract | 5,314,648 |
| 3 | "Framework"[Title/Abstract] OR "Model"[Title/Abstract] |  | 2,521,755 |
| 1 + 2 + 3 | "diarrh*"[Title/Abstract] AND ("prevent*"[Title/Abstract] OR "control*"[Title/Abstract]) AND ("Framework"[Title/Abstract] OR "Model"[Title/Abstract]) | Title/abstract | 2,761 |
| **Total** |  |  | **4227** |
| **Scopus** |  |  |  |
| 22^nd^ April 2021 | TITLE-ABS-KEY (waterborne AND framework): waterborne framework | article title, abstract, keywords | 290 |
| 22^nd^ April 2021 | TITLE-ABS-KEY (waterborne AND model) waterborne model | article title, abstract, keywords | 1,988 |
| 2^nd^ August 2021  1 | TITLE (diarrh*) | Title | 36810 |
| 2^nd^ August 2021  2 | TITLE (framework OR model) | Title | 2423298 |
| 2^nd^ August 2021  1 + 2 | (TITLE (diarrh* ) )  AND  ( TITLE ( framework  OR  model ) ) |  | 363 |
| **Total** |  |  | **2641** |
| **Web of Science** |  |  |  |
| 22^nd^ April 2021 | Waterborne framework (Waterborne framework) | Topic | 224 |
| 22^nd^ April 2021 | Waterborne model | Topic: title, abstract, author keywords, and Keywords PLUS | 1889 |
| 2^nd^ August 2021  1 | Diarrh***** (Title) |  | [30,971](https://www-webofscience-com.uplib.idm.oclc.org/wos/woscc/summary/921e3583-98fa-4575-95bb-f08fd7ce0b39-02eb2439/relevance/1) |
| 2 | Framework OR Model (Title) |  | [2,803,212](https://www-webofscience-com.uplib.idm.oclc.org/wos/woscc/summary/2bc2dc64-8168-45f7-b004-d982e8cc6905-02eb262a/relevance/1) |
| 3 | 1 and 2 |  | 462 |
| **Total** |  |  | 2575 |
| **Grand total** |  |  | **9443** |
