# Supplementary material for: Frameworks for mitigating the risk of waterborne diarrheal diseases: A scoping review
Source: PLoS One. 2022 Dec 9;17(12):e0278184. doi: 10.1371/journal.pone.0278184 (PMC9733858; doi:10.1371/journal.pone.0278184)
Supplement: S1 File — (DOCX) [file pone.0278184.s003.docx]

**INCLUDED AND EXCLUSDED LITERATURE WITH REASONS**

**INCLUDED LITERATURE**

1. Kleinau E, Post M, Rosensweig F: Advancing hygiene improvement for diarrhea prevention: Lessons learned. *Strategic Report* 2004, 10.
2. Bateman OM, Jahan RA, Brahman S, Zeitlyn S, Laston SL: Prevention of diarrhea through improving hygiene behaviors. *The Sanitation and family Education (SAFE) Pilot Project Experience,(42)* 1995.
3. Kar K., Chambers R. Handbook on community-led total sanitation. 2008.
4. WHO. PHAST Step-by-Step Guide: a participatory approach for the control of diarrheal disease. World Health Organization 1998.
5. WHO/UNICEF. Ending preventable child deaths from pneumonia and diarrhea by 2025: The integrated Global Action Plan for Pneumonia and Diarrhea (GAPPD), World Health Organization 2013.

**REASONS FOR EXCLUSION OF LITERATURE**

**NO DIARRHEA OUTCOME TARGET OR PROPER DIARRHEA OUTCOME**

1. Sarkar A: The role of new ‘smart technology’ to provide water to the urban poor: a case study of water ATMs in Delhi, India. *Energy, Ecol Environ* 2019, 4(4):166-174.
2. Zheng H, Negenborn RR, Lodewijks G: Robust Distributed Predictive Control of Waterborne AGVs - A Cooperative and Cost-Effective Approach. *IEEE Transactions on Cybernetics* 2018, 48(8):2449-2461.
3. Branco M, Saito CH: Waterborne Disaster Prevention in Brazil: Assessing the Level of Implementation of the Hyogo Framework for Action. *Desenvolvimento E Meio Ambiente* 2017, 42:180-201.

**NO PROPER FRAMEWORK / MATHEMATIC FRAMEWORK / INTERVENTION**

**STUDIES ONLY**

1. Collins OC, Duffy KJ: Mathematical Analyses on the Effects of Control Measures for a Waterborne Disease Model with Socioeconomic Conditions. *J Comput Biol*.
2. <https://www.hindawi.com/journals/jam/2018/2528513/>
3. Dabbagh L, Green LW, Walker GM: Case study: application of precede and proceed as a framework for designing culturally sensitive diarrhea prevention programs and policy in arab countries. Int Q Community Health Educ 1991, 12(4):293-315.
4. Taylor SM, Hayes M, Frank J, White N: The risk approach in diarrheal disease intervention in Grenada. Soc Sci Med 1990, 30(10):1089-1095.
5. Taylor CE, Greenough WB, 3rd: Control of diarrheal diseases. Annu Rev Public Health 1989, 10:221-244.
6. Pérez-Cuevas R, Guiscafré H, Romero G, Rodríguez L, Gutiérrez G: Mothers' health-seeking behaviour in acute diarrhoea in Tlaxcala, Mexico. *J Diarrhoeal Dis Res* 1996, 14(4):260-268.
7. Bailey JE, Coombs DW: Effectiveness of an Indonesian model for rapid training of Guatemalan health workers in diarrhea case management. *J Community Health* 1996, 21(4):269-276.
8. Jose MV, Bobadilla JR: Epidemiological model of diarrhoeal diseases and its application in prevention and control. *Vaccine* 1994, 12(2):109-116.
9. Stanton B, Black R, Engle P, Pelto G: Theory-driven behavioral intervention research for the control of diarrheal diseases. *Soc Sci Med* 1992, 35(11):1405-1420.
10. Bentley ME: Household behaviors in the management of diarrhea and their relevance for persistant diarrhea. *Acta Paediatr* 1992, 81 Suppl 381:49-54.
11. Ferreira DC, Graziele I, Marques RC, Gonçalves J: Investment in drinking water and sanitation infrastructure and its impact on waterborne diseases dissemination: The Brazilian case. *Sci Total Environ* 2021, 779:146279.
12. Affandi P, Salam N: Optimal Control of diarrhea Disease model with Vaccination and Treatment. In*: 2021*; 2021.
13. Yłldłz TA: Optimal control problem of a non-integer order waterborne pathogen model in case of environmental stressors. *Front Phys* 2019, 7(JULY).
14. Jalba DI, Cromar NJ, Pollard SJT, Charrois JW, Bradshaw R, Hrudey SE: Safe drinking water: Critical components of effective inter-agency relationships. *Environ Int* 2010, 36(1):51-59.
15. Batterman S, Eisenberg J, Hardin R, Kruk ME, Lemos MC, Michalak AM, Mukherjee B, Renne E, Stein H, Watkins C: Sustainable control of water-related infectious diseases: a review and proposal for interdisciplinary health-based systems research. *Environ Health Perspect* 2009, 117(7):1023-1032.
16. Organization WH: Combating waterborne disease at the household level. 2007.
17. Eisenberg JN, Scott JC, Porco T: Integrating disease control strategies: balancing water sanitation and hygiene interventions to reduce diarrheal disease burden. *Am J Public Health* 2007, 97(5):846-852.
18. Gao W, Li G, Liu X, Yan H: The impact of "Child Care" intervention in rural Primary Health Care Program on prevalence of diarrhea among children less than 36 months of age in rural western China. *BMC Pediatr* 2018, 18(1):228.
19. Okafor CE, Ekwunife OI: Cost-effectiveness analysis of diarrhoea management approaches in Nigeria: A decision analytical model. *PLoS Negl Trop Dis* 2017, 11(12).
20. Mumford JE, Flaxman AD, Deason AW, Abdel-Messih IA, Shonka T, Brown J, Weaver M, Mokdad AH: Cost-effectiveness of interventions to prevent diarrhea: introducing a microsimulation model for children under five in every country from 2005 to 2015. *Am J Trop Med Hyg* 2017, 97(5):546-546.
21. Monis P, Lau M, Harris M, Cook D, Drikas M: Risk-based management of drinking water safety in Australia: Implementation of health based targets to determine water treatment requirements and identification of pathogen surrogates for validation of conventional filtration. *Food Waterborne Parasitol* 2017, 8-9:64-74.
22. Santos J, Pagsuyoin SA, Latayan J: A multi-criteria decision analysis framework for evaluating point-of-use water treatment alternatives. *Clean Technol Environ Policy* 2016, 18(5):1263-1279.
23. Rietveld LC, Siri JG, Chakravarty I, Arsenio AM, Biswas R, Chatterjee A: Improving health in cities through systems approaches for urban water management. *Environmental Health* 2016, 15.
24. Collins OC, Duffy KJ: Optimal control intervention strategies using an n-patch waterborne disease model. *Nat Resour Model* 2016, 29(4):499-519.
25. Bosomprah S, Beach LB, Beres LK, Newman J, Kapasa K, Rudd C, Njobvu L, Guffey B, Hubbard S, Foo K *et al*: Findings from a comprehensive diarrhoea prevention and treatment programme in Lusaka, Zambia. *BMC Public Health* 2016, 16:475.
26. Zheng H, Negenborn RR, Lodewijks G: Model predictive control of a waterborne AGV at the operational level. In: *Maritime-Port Technology and Development - Proceedings of the International Conference on Maritime and Port Technology and Development, MTEC 2014: 2015*; 2015: 99-108.
27. Popoola T, Mchunu G: Application of PRECEDE‐PROCEED model to tackle problems identified with diarrhoea burden among under‐5s in B otswana. *Int J Nurs Pract* 2015, 21:67-70.
28. Nicole W: The WASH approach: Fighting waterborne diseases in emergency situations. In*.*: NLM-Export; 2015.
29. Walker CLF, Walker N: The Lives Saved Tool (LiST) as a model for diarrhea mortality reduction. *BMC Med* 2014, 12.
30. Sokolova E, Pettersson TJR, Bergstedt O, Hermansson M: Hydrodynamic modelling of the microbial water quality in a drinking water source as input for risk reduction management. *Journal of Hydrology* 2013, 497:15-23.
31. Misra A, Singh V: A delay mathematical model for the spread and control of water borne diseases. *J Theor Biol* 2012, 301:49-56.
32. Gupta N, Mutukkanu T, Nadimuthu A, Thiyagaran I, Sullivan-Marx E: Preventing waterborne diseases: analysis of a community health worker program in rural Tamil Nadu, India. *J Community Health* 2012, 37(2):513-519.
33. Devipriya G, Kalaivani K: Optimal control of multiple transmission of water-borne diseases. *International Journal of Mathematics and Mathematical Sciences* 2012, 2012.

**LOOKED AT WATER SUPPLY MANAGEMENT/ TARGET NOT DIARRHEA PREVENTION BUT GENERAL WATER TREATMENT**

1. <https://www.who.int/publications/i/item/9789241548427>
2. Eheart JW, Brill ED, Lence BJ, Kilgore JD, Uber JG: Cost efficiency of time‐varying discharge permit programs for water quality management. Water Resour Res 1987, 23(2):245-251.
3. Rizak S, Cunliffe D, Sinclair M, Vulcano R, Howard J, Hrudey S, Callan P: Drinking water quality management: A holistic approach. *Water Sci Technol* 2003, 47(9):31-36.
4. Momba MNB, Makala N, Tyafa Z, Brouckaert BM: A model partnership for sustainable production of safe drinking water for rural communities in South Africa. *South African Journal of Science* 2005, 101(7-8):335-336.
5. Njemanze PC, Anozie J, Ihenacho JO, Russell MJ, Uwaeziozi AB: Application of risk analysis and geographic information system technologies to the prevention of diarrheal diseases in Nigeria. *The American journal of tropical medicine and hygiene* 1999, 61(3):356-360.
6. McKie A, Bartram J, Colbourne J, Clarke B, Theobalds A: Framework for drinking-water safety in Saint Lucia health based targets. In: *Sustainable Development of Water Resources, Water Supply and Environmental Sanitation: Proceedings of the 32nd WEDC International Conference: 2007*; 2007: 474-481.
7. Petterson SR: Application of a QMRA Framework to Inform Selection of Drinking Water Interventions in the Developing Context. *Risk Anal* 2016, 36(2):203-214.
8. Gunnarsdottir MJ, Gardarsson SM, Bartram J: Developing a national framework for safe drinking water—Case study from Iceland. *Int J Hyg Environ Health* 2015, 218(2):196-202.
9. Hasan TJ, Hicking A, David J: Empowering rural communities: Simple Water Safety Plans. *Water Science and Technology: Water Supply* 2011, 11(3):309-317.
10. Jenkins BR: Management of waterborne disease. In: *Global Issues in Water Policy.* vol. 19; 2018: 277-310.
11. Bereskie T, Rodriguez MJ, Sadiq R: Drinking Water Management and Governance in Canada: An Innovative Plan-Do-Check-Act (PDCA) Framework for a Safe Drinking Water Supply. *Environ Manage* 2017, 60(2):243-262.
12. Nare L, Odiyo J, Francis J, Potgieter N: Framework for effective community participation in water quality management in Luvuvhu Catchment of South Africa. *Physics and Chemistry of the Earth, Parts A/B/C* 2011, 36(14-15):1063-1070.

**OTHER REASONS**

**NUTRITIONAL INTERVENTION OR ANIMALS STUDIES**

1. Elder J, Touchette P, Smith W, Geller S, Foote D: The healthcom project and the behavioral management of diarrhea. *Int Q Community Health Educ* 1987, 8(3):201-212.
2. Whitley L, Hutchings P, Cooper S, Parker A, Kebede A, Joseph S, Butterworth J, Van Koppen B, Mulejaa A: A framework for targeting water, sanitation and hygiene interventions in pastoralist populations in the Afar region of Ethiopia. *Int J Hyg Environ Health* 2019, 222(8):1133-1144.

**PAPER NOT RELATED TO THE TOPIC**

1. Okafor CE, Ekwunife OI: Cost-Effective Approach in Management of Diarrhoea in Nigeria: A Decision Analytical Model. *Pharmacoepidemiol Drug Saf* 2016, 25:622
2. Zeilhofer P, Zeilhofer L, Hardoim EL, de Lima ZM, Oliveira CS: GIS applications for mapping and spatial modeling of urban-use water quality: a case study in District of Cuiaba, Mato Grosso, Brazill. *Cad Saude Publica* 2007, 23(4):875-884.

**NO FULL STUDY FOUND**

1. Ford T, Hamner S: Control of Waterborne Pathogens in Developing Countries. *Environ Microbiol* 2010:33-56.
2. Malapane TA: Water and Health in Limpopo: Implementing a Safe, Sustainable Water Supply System; 2011.

**PROPOSALS NO FRAMEWORK**

1. McIntyre KM, Bolton FJ, Christley RM, Cleary P, Deja E, Durie AE, Diggle PJ, Hughes DA, de Lusignan S, Orton L *et al*: A Fully Integrated Real-Time Detection, Diagnosis, and Control of Community Diarrheal Disease Clusters and Outbreaks (the INTEGRATE Project): Protocol for an Enhanced Surveillance System. *JMIR Res Protoc* 2019, 8(9):e13941.
2. Kang JY, Aldstadt J: Examining time-dependent effects of water, sanitation, and hygiene (WASH) interventions using an agent-based model. *Trop Med Int Health* 2019, 24(8):962-971.
