# Supplementary material for: Frameworks for mitigating the risk of waterborne diarrheal diseases: A scoping review
Source: PLoS One. 2022 Dec 9;17(12):e0278184. doi: 10.1371/journal.pone.0278184 (PMC9733858; doi:10.1371/journal.pone.0278184)
Supplement: Attachment — Submitted filename: Response to Reviewers.docx [file pone.0278184.s004.docx]

**FEEDBACK TO REVIEW COMMENTS**

| **COMMENT** | **FEEDBACK** |
| --- | --- |
| **REVIEWER 1** |  |
| lines 18 and 19: health promotion and 19 education, and enabling environment and clinical treatments  Comment: and must not be repeated | Thank you for the feedback. One ‘and’ has been removed line 45 |
| Line 37 to 39 Waterborne diarrheal diseases include cholera, campylobacteriosis, typhoid fever, salmonellosis, shigellosis/ bacillary dysentery, cholera yersinosis, cryptosporidiosis, amebiasis (amoebic dysentery), cyclosporiasis and giardiasis Comment: where are viral causes | Thank you for this feedback. The viral causes have been added as advised lines 67 to 70 |
| line 74: This scoping review summarizes the Comment: This sentence needs to be re-written | Thank you. The objective of the review has been rewritten. Lines 106 to 108 |
| Line 78: Scoping reviews are appropriate to identify and map existing evidence, identify key Comment: This sentence needs to re-written | Thank you for the comment. The sentence has been rewritten. Lines 112 to 113 |
| Line 125: We used a data charting form to extract data from selected articles. We modified the Comment: to writ we......., we.....  This type of writing needs to be changed | Revised thank you. Lines 166 to 168 |
| Line 251: . The approach was developed in Bangladesh by WaterAid. Comment: needs more details | Thanks. Details have been included. Lines 303 to 305 |
| Line 298: • Develop a clear country-level strategy and work plan, with assigned key responsibilities: Comment: Please, write the same issue in the manuscript using the same manner | Thank you for this commnet. The proposed framework is developed for use in Zambia and similar set up as indicated in the proposed framework lines 588 to 590 |
| Lines 387 and 388: We scored the five frameworks included in this scoping review (Table 4) using the 17 criteria outlined in Table 2. We identified the following strengths in all the frameworks. All the Comment: in all parts of the manuscript, english needs to be improved which We.....we....we The use of We in the first of all sentences needs to be changed. | The language in all parts of the manuscript has been revised. Please kindly check the whole manuscript. |
| Lines 485 to 488: Conclusion We reviewed frameworks for mitigating the risk of diarrheal diseases. No framework specifically addressed waterborne diarrheal diseases thus we included frameworks aiming to mitigate diarrheal diseases in general. Comment: Please, write the conclusions directly. English language in all the manuscript needs to be revised and the method of writing needs to be revised. The structure of the manuscript and the method authors expressed the data needs to be revised. | Thank you for this comment. The conclusion has been modified as recommended check lines 610 to 627  Kindly note that the whole manuscript has been edited. |
| **REVIEWER 2** |  |
| Authors have summarized frameworks related to mitigation of diarrheal diseases, focusing on waterborne diarrhoea. It may also be important to analyse/ incorporate the outcomes of implementing these frameworks in the evaluation scoring system, if such data are available. It may also provide insights on the weaknesses of these frameworks. | Thank you for this recommendation. The outcomes for the implementation of frameworks have been added in the results section not necessarily in the scoring system but in a descriptive way please check lines 462 to 478 we have also added related information in the discussion |
| Authors have provided very few suggestions for developing future frameworks to minimize the waterborne diarrhoea (line 450-465). This section needs to be expanded using examples from the selected frameworks, other studies, and author’s own opinions. | Thank you for this comment. More information of the future framework have been provided lines 551 to 590 |
| Authors have highlighted the unavailability of electronic data collection in the manuscript several times. However, several studies were conducted in a time where electronic devices are not as common as today and digital literacy is not really high. Therefore, it may not be directly highlighted as a weakness. | Thank you for this valuable comment. We have not removed the electronic means of data collection, tranfers and storage in the scoring criteria. However, we have included it under the limitation section considering the comment given. Check lines 598 to 603 |
